# Complete Genome Sequence of Brevibacillus laterosporus Bl-zj, an Algicidal Bacterium Isolated from Soil

**DOI:** 10.1128/MRA.00408-19

**Published:** 2019-07-25

**Authors:** Jian Cai, Dianyu Chen, Dong Chen, Xianghu Huang, Changling Li, Huiling Liu, Mengjie Li, Guanbao Li, Yulei Zhang

**Affiliations:** a Fisheries College of Guangdong Ocean University, Zhanjiang, China; b Shenzhen Institute of Guangdong Ocean University, Shenzhen, China; c Engineering Technology Research Center for Algae Breeding and Application of Guangdong Province, Zhanjiang, China; Indiana University, Bloomington

## Abstract

Brevibacillus laterosporus can be used as a biocontrol agent for varieties of plants, as it is a pathogen of invertebrates and can also inhibit many bacteria and fungi. Here, we describe the complete genome sequence of B. laterosporus strain Bl-zj, an algicidal bacterium on cyanobacteria isolated from the soil in China.

## ANNOUNCEMENT

Brevibacillus laterosporus, first discovered in 1912 (1), is a Gram-positive, rod-shaped, and facultatively anaerobic bacterium that produces a canoe-shaped parasporal body (CSPB) tightly attached to one side of the spore (2, 3). B. laterosporus was reported as a pathogen of invertebrates and an inhibitor of some bacteria and fungi, in which the extent of virulence was determined by the size and components of its spores and the specific extracellular metabolites (4–6). *B. laterosporus* strain Bl-zj was isolated from an intertidal zone soil sample in Zhanjiang, China (7), and was shown to have algicidal activity and to indirectly dissolve algae by secreting peptides (8). Sequencing, assembly, and annotation of the *B. laterosporus* Bl-zj genome provide valuable information for us to identify alga-dissolving substances and understand its algicidal mechanisms.

*B. laterosporus* Bl-zj was cultured on LB medium at 30°C. Genomic DNA was isolated using the TIANamp bacterial DNA kit and fragmented using a Covaris instrument. End repairing, adapter ligation, and purification were performed on 5 μg of fragmented DNA using the SMRTbell template prep kit (PacBio) for library preparation. The final library was quantified and the size determined via a Qubit fluorometer and Agilent Bioanalyzer 2100. The single-molecule real-time (SMRT) sequencing work was conducted using the PacBio RS II system. Subreads with a readScore of ≥0.75 were reserved using the SMRT Analysis software for quality control. After filtering of raw data, 142,912 cleaned subreads (*N*_50_, 17,369 bp) totaling 1,899,845,317 bp were obtained, with an average length of 13,294 bp. The subreads were assembled using the Canu software (version 1.5) (9), followed by Minimus2 for genome circularization (10); finally, a complete genome circle was confirmed by the assembly alone. The DNA sequence was submitted to the NCBI Prokaryotic Genomes Automatic Annotation Pipeline (PGAAP) for annotation. The amino acid sequences from each open reading frame were used as input to the COG (11), KEGG pathway (12), GO (13), and nr protein functional databases with metabolic pathway enrichment analysis, and then the annotated genome was submitted to GenBank.

The complete genome of *B. laterosporus* consists of 5,202,546 bp, with an average GC content of 41.33%. A total of 4,594 predicted protein-coding genes were identified, with an 84.21% coding rate and average length of 954 bp. The chromosome also contained 36 rRNA genes, 112 tRNA genes, 76 noncoding RNA (ncRNA) genes, and 5 pseudogenes. For the protein-coding genes, 4,545 (98.93%), 3,047 (66.33%), 2,079 (45.25%), and 2,153 (46.87%) genes were assigned functional categories from the nr, COG, KEGG pathway, and GO databases, respectively. For a better understanding of the algicidal activity of *B. laterosporus* Bl-zj as reflected in the genome, the virulence genes, secretory proteins, transporters, and pathogen-host interaction factors were further predicted and analyzed. As expected, epsilon toxin, mosquitocidal toxin, alveolysin, lethal factor, chitinase, microbial collagenase, and various carbohydrate hydrolases and peptidases were identified, which are important virulence characteristics of bacteria in invasion and infection, as well as algicidal responses (14–18).

### Data availability.

The complete genome sequence has been deposited in GenBank under accession number CP032848. The SMRT sequence raw data have been deposited in the NCBI Sequence Read Archive under BioProject accession number PRJNA494917.
